# 241. A Comparison of Staphylococcal and Streptococcal Septic Arthritis with Lyme Arthritis

**DOI:** 10.1093/ofid/ofab466.443

**Published:** 2021-12-04

**Authors:** Don Kannangara, Dhyanesh Pandya

**Affiliations:** St Luke’s University Health Network, Bethlehem, Pennsylvania

## Abstract

**Background:**

Septic arthritis is considered the most important differential diagnosis in suspected Lyme arthritis. The present study sheds light on the most frequent misdiagnoses in Lyme arthritis cases and clues for differentiation from Staphylococcal and Streptococcal septic arthritis.

**Methods:**

We studied patients with positive joint fluid cultures with *Staphylococcus aureus* (SA) and streptococci and Lyme polymerase chain reaction (PCR) positive joint fluid in *9* hospitals in Eastern Pennsylvania and 1 in Warren county, New Jersey over a 3 year period.

**Results:**

One hundred and thirty four out of 7000 SA and 21 out of 1321 streptococcal isolates were from joint fluid. Twenty nine had Lyme arthritis, ages 5-74 ( 24 males,5 females). Twelve out of 29 were ages 10-18 with 20/29 under age 40. Predominant joint affected was a single knee 27/29. All had pain with or without swelling and little erythema. Two had fever. One reported a tick bite. None had other manifestations of Lyme disease. The diagnosis at the initial visit was sprain or sports injury in 5, osteoarthritis in 5, inflammatory arthritis or gout in 2 each, i septic arthritis, 1 viral syndrome and 1 ruptured Baker's cyst. Joint fluid count range was 3500-77,360 with only 3 over 50,000. White blood cell count (wbc) range was 3200-14,580. SA arthritis involved knee in 66 (49.3%), hip 31(23.9%), elbow 19 (14.2%), shoulder 14 (10.4%) with 2 wrist, 1 ankle and 1 sterno-clavicular joint. Fifty seven had a history of joint surgery. Eighty six were male and 48 female. age range 14-95 with a median age 65. Synovial fluid cell count was 335-470,000 and wbc 5,200-28,410 . Streptococcal septic arthritis ( 13 male 8 female) involved the knee in 17/21 with one each of hip, elbow, shoulder. The ages were 36-86 with 15/21 over age 60. Synovial fluid count was15,242-641,425 . Wbc count 11,140-25,080 .Nine out 21 had prior joint surgery.

**Conclusion:**

Lyme arthritis patients were younger, mostly involving 1 knee, majority male without other manifestations of Lyme disease. Highest synovial fluid count was 77,360 and highest wbc count 14,580. Most frequent misdiagnoses were sports injury/sprain or osteoarthritis. SA and Streptococcal arthritis were mostly in elderly, with higher joint fluid cell and wbc counts. Only 1/29 Lyme arthritis was initially misdiagnosed septic arthritis.

**Disclosures:**

**All Authors**: No reported disclosures

